# Clinical significance of urinary exosomal microRNAs in patients with IgA nephropathy

**DOI:** 10.1038/s41598-023-44460-5

**Published:** 2023-10-11

**Authors:** Soo-Young Yoon, Jin Sug Kim, Su Woong Jung, Yang Gyun Kim, Hyeon Seok Hwang, Ju-Young Moon, Sang-Ho Lee, Jung-Woo Seo, Junhee Seok, Donghyun Tae, Kyunghwan Jeong

**Affiliations:** 1 Division of Nephrology, Department of Internal Medicine, Kyung Hee University College of Medicine, Kyung Hee University Hospital, 23 Kyungheedae-Ro, Dongdaemun-Gu, Seoul, 02447 Republic of Korea; 2 Division of Nephrology, Department of Internal Medicine, Kyung Hee University College of Medicine, Kyung Hee University Hospital at Gangdong, Seoul, Korea; 3 Core Research Laboratory, Medical Science Institute, Kyung Hee University Hospital at Gangdong, Seoul, Korea; 4 School of Electrical Engineering, Korea University, Seoul, Korea

**Keywords:** Computational biology and bioinformatics, Nephrology

## Abstract

Immunoglobulin A nephropathy (IgAN) is the most common primary glomerulonephritis worldwide. The clinical relevance of 11 urinary exosomal microRNAs (miRNAs) was evaluated in patients with IgAN. From January 2009 to November 2018, IgAN (n = 93), disease control (n = 11), and normal control (n = 19) groups were enrolled. We evaluated the expression levels of urinary exosomal miRNAs at the baseline and their relationship with clinical and pathologic features. This study aimed to discriminate statistically powerful urinary exosomal miRNAs for the prognosis of IgAN. Urinary miRNA levels of miR-16-5p, miR-29a-3p, miR-124-3p, miR-126-3p, miR-199a-3p, miR-199b-5p, and miR-335-3p showed significant correlation with both estimated glomerular filtration rate (eGFR) and urine protein-to-creatinine ratio (uPCR). In univariate regression analysis, age, body mass index, hypertension, eGFR, uPCR, Oxford classification E, and three miRNAs (miR-16-5p, miR-199a-3p, and miR-335-3p) were associated with disease progression in patients with IgAN. The area under the curve (AUC) of miR-199a-3p was high enough (0.749) without any other clinical or pathologic factors, considering that the AUC of the International IgAN Risk Prediction Tool was 0.853. Urinary exosomal miRNAs may serve as alternative prognostic biomarkers of IgAN with further research.

## Introduction

Immunoglobulin A nephropathy (IgAN) is a leading cause of chronic kidney disease (CKD) and the most common cause of primary glomerulonephritis worldwide. The prognosis of patients with IgAN varies, and approximately 15–40% of patients develop end-stage kidney disease (ESKD) within 20 years^1^. Therefore, early diagnosis, risk prediction for progressive CKD, and proper time management are important for IgAN.

Exosomes (40–130 nm) are bilayer membrane-derived vesicles derived from the endocytic lipid compartment and can be extracted from urine and various body fluids, including serum, plasma, and saliva^2,3^. Exosomes play a crucial role in cell-to-cell communication as they can carry proteins, nucleotides, deoxynucleotides, and microRNAs (miRNAs) to mediate cell proliferation, differentiation, etc.^4^. MiRNAs are a group of short (18–22 nucleotides), small, noncoding RNAs that post-transcriptionally regulate gene expression. Since extracellular vesicles detected in urine samples are from glomerular podocytes, tubular epithelial cells, and tubular endothelial cells, exosomes from extracellular vesicles in urine can inform researchers of the status of kidney-related diseases^5,6^. Therefore, the urinary expression changes in miRNAs as an extracellular vesicle components has a potential to be used as a novel biomarker for early diagnosis of various kidney diseases including IgAN^6^.

Early diagnosis and optimal therapeutic strategies IgAN are important, and the development of promising prognostic biomarkers for IgAN is desired. Several previous studies have shown that miRNAs are associated with common aspects of kidney disease, including fibrosis and inflammation, which are related to downstream pathways operating in IgAN^7^. MiRNAs have been suggested as potential disease-specific biomarkers for predicting disease activity and prognosis. However, further studies are needed because most of the previous studies involved a relatively small sample size, and the connection between a variety of miRNAs and related genes has not been studied.

Urinary miRNAs are likely involved in several pathways of IgAN pathogenesis, and several miRNAs and their targets comprise a complex network involved in the pathogenesis of IgAN. This study aimed to identify novel miRNAs that enable anticipation of IgAN progression, which could contribute to the interpretation of the clinical relevance of these urinary exosomal markers as predictors of renal prognosis.

## Methods

### Study design and urine sample collection

**Figure 1:**
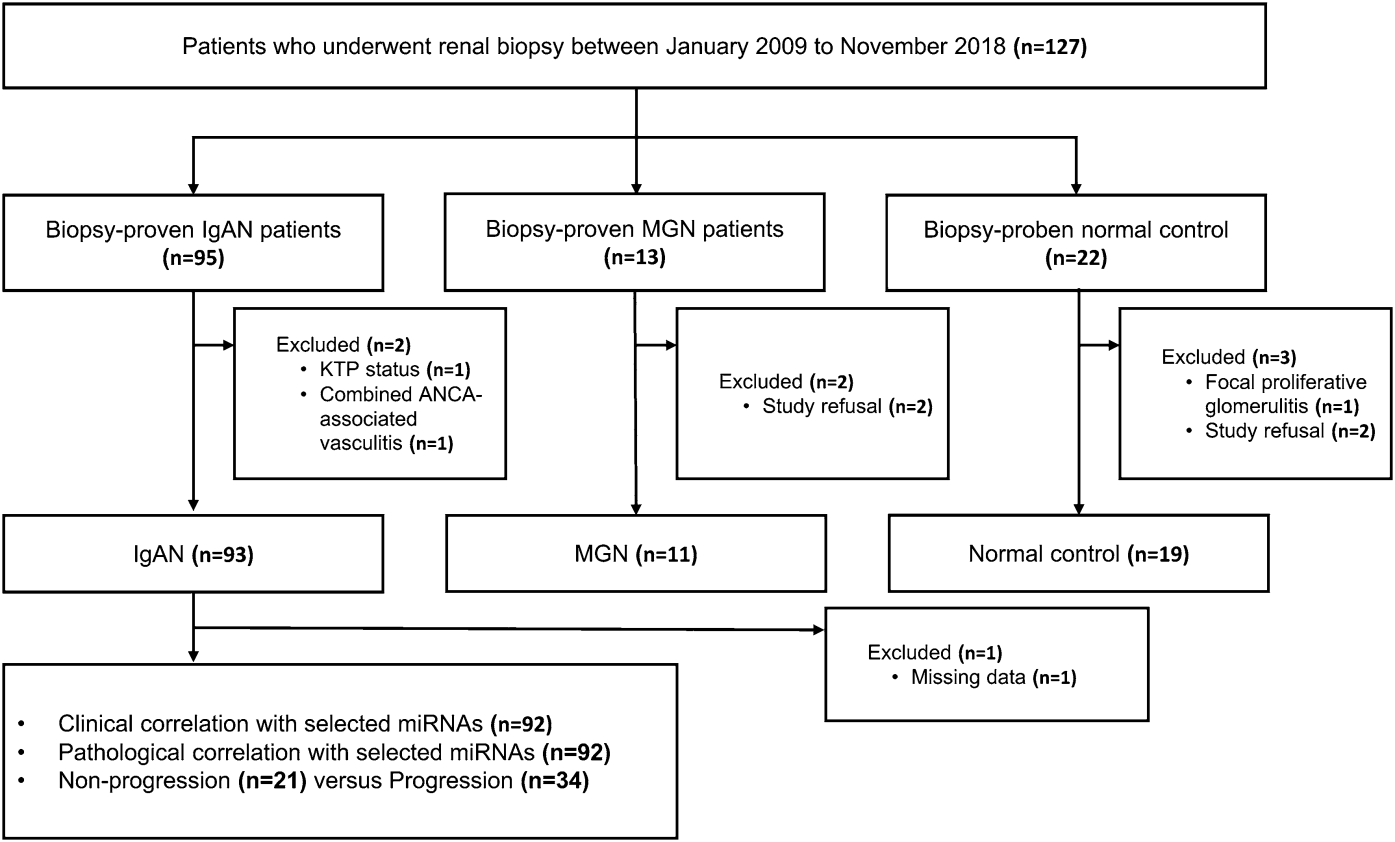
Flow chart of the population selection. IgAN, IgA nephropathy; MGN, membranous glomerulonephritis.

Among 127 subjects in this study, we finally recruited 93 patients with biopsy-proven IgAN in a cross-sectional sample collection study from January 2009 to November 2018 (Fig. 1). Patients with non-IgAN nephropathy were also included as disease controls: 11 patients with membranous glomerulonephritis (MGN). Nineteen subjects without kidney disease were included as the controls.

Baseline variables, including age, sex, body mass index (BMI), and medications that were taken before renal biopsy, were recorded. Blood samples were obtained for the measurement of serum albumin, creatinine, and IgA, and midstream void morning urine samples were collected to assess the amount of proteinuria and presence of hematuria at the time of renal biopsy. Renal function was assessed via estimated glomerular filtration rate (eGFR) using the Chronic Kidney Disease Epidemiology Collaboration (CKD-EPI) equation^8^. The amount of proteinuria was calculated using urine protein-to-creatinine ratio (uPCR). Urine samples were centrifuged at 2,000×g at room temperature for 20 min to separate the pellets and supernatants, and were stored at − 80 °C until use. Urinary miRNAs were extracted from the exosomes, and then each miRNA expression level was calculated following the protocol.

All the study procedures complied with the ethical guidelines of the Helsinki Declaration of 1975, revised in 2008, and were approved by the Institutional Review Board of Kyung Hee University Hospital. The approval number from Kyung Hee University Hospital was 2022-12-040, and written informed consent was obtained from all participants.

### Measurement of clinical outcome

Patients with biopsy-proven IgAN who experienced less than a 10% change in eGFR for a period of over five years following diagnosis were categorized as "non-progressors." On the other hand, individuals classified as "progressors" had an eGFR decrease more than 50% within five years since diagnosis or required renal replacement therapy. IgAN biopsies were scored according to the Oxford classification system, which encompasses five histopathological characteristics (M, E, S, T, and C scores)^9^. The International IgAN Risk Prediction Tool (IIgANRPT) is a tool that predicts the risk of progression based on a eGFR at the biopsy date, mean arterial blood pressure, amount of proteinuria, age at the biopsy date, Oxford Classification score, renin angiotensin system (RAS) blocker, or immunosuppression usage, and race. Subject with missing data was excluded from the regression analysis.

### Prediction and quantification of urinary exosomal miRNAs

To search for urinary miRNA candidates for IgAN, we performed enrichment tests between the significantly expressed mRNAs and miRNA target gene sets. First, we searched the keywords “IgA nephropathy” and “glomerulus” or “IgA nephropathy” and “tubulointerstitium” in the GEO database to select candidate mRNAs. We ensured that any datasets with overlapping samples between the glomerulus and tubulointerstitium groups were excluded from our selection process. Five datasets for glomerulus (GSE104948, GSE93798, GSE99339, GSE50469, and GSE37460) and five datasets for tubulointerstitium (GSE104954, GSE99340, GSE99325, GSE35488, and GSE35487) that had the whole gene expression profiles of both IgAN patients and healthy controls (Supplementary Table 1). We used pre-calculated log2-based gene expression indices from the original papers as provided in the database. Since all of these datasets were obtained from the same Affymetrix GeneChip platform, we were able to merge them easily using the same method. Probesets were converted to gene symbols following the Affymetrix GeneChip annotation. In cases where one gene was measured by multiple probesets, we selected the one with the largest variance to represent the gene. As a result, the expression measurements of approximately 10,000 genes that were common to all datasets were included in the subsequent meta-analysis.

Second, we conducted a meta-analysis of these datasets using the GeneMeta R package, following the approach by Choi et al. to identify genes that were significantly different between patients IgAN and healthy controls^10^. Although the details were described in Choi et al., we provided a brief explanation of the analysis steps here. This method calculated the effect size of each dataset in a two-sample test setting using standardized-mean difference^11^. Subsequently, the effect sizes of multiple datasets were modeled using a random effect model. In the random effect model, ${y}_{i}\sim N({\theta }_{i},{s}_{i}^{2})$ and ${\theta }_{i} \sim N(\mu , {\tau }^{2})$ where ${y}_{i}$ is the effect size of dataset $i$. Here, the parameters $\mu$ and $\tau$ were estimated using the standard approach of the random effect model^12^. For the multiple datasets, the z-score was computed as the average effect size for each gene. The statistical significance of z-scores are then estimated by adapting the significance analysis of microarray approach^13^. Column-wise permutation was introduced within each dataset, and the null distribution of z-scores was empirically determined. The false discovery rate (FDR) of a gene was calculated by counting the number of permuted cases that showed more extreme z-scores than expected. The overall fold change (FC) of a gene was also computed as the geometric mean of the fold changes observed in multiple datasets. The analysis yielded significantly expressed mRNAs with a FDR < 0.05 and a FC > 1.5. FDRs were obtained from 1000 permutations using multiple hypothesis tests. In total, we found 884 and 67 significant genes from glomerulus and tubulointerstitium datasets, respectively (Supplementary Table 2 and 3).

Third, miRNA target gene sets were downloaded from three databases, miRTarBase (https://mirtarbase.cuhk.edu.cn/), TargetScan (http://www.targetscan.org/vert_72/), and miRDB (http://www.mirdb.org/); 1,110,357 target genes against 1802 miRNAs were extracted. We then selected pairs of miRNAs and target genes that were found in multiple databases. In total, we extracted 405,092 genes linked to 1760 miRNAs. These miRNA-gene pairs represented the expected genes whose expression was perturbed by the corresponding miRNAs. Therefore, miRNAs whose target genes were dominantly found among the significant genes from the meta-analysis could be considered of particular interest.

Finally, we selected significantly activated or suppressed miRNAs by conducting enrichment tests on significantly expressed mRNAs within miRNA gene sets. Fisher’s exact test was employed for these enrichment tests. Assuming that the meta-analysis found *n* significant genes, a miRNA had *m* target genes, and they had *k* overlapping genes, the Fisher’s exact test was then performed on the observed cross-table of $[k, n-k, m-k, N-n-m+k]$, where *N* was the total number of genes whose expression was measured. Essentially, this test evaluated the significance of the overlap compared to what would be expected if the two sets were assumed to be independent. The p-values of miRNAs were calculated based on the hypergeometric distribution. The FDRs were determined from the p-value distribution. With FDR < 0.05, five miRNAs were selected from glomerular-related genes, and six miRNAs were chosen from tubulointerstitial-related genes. As a result, 11 candidate urinary miRNAs were selected: miR-16-5p, miR-26b-3p, miR-29a-3p, miR-29c-3p, miR-124-3p, miR-126-3p, miR-199a-3p, miR-199b-5p, miR-335-3p, miR-615-3p, and miR-29b-3p.

Urinary miRNAs were extracted from the exosomes, as described below. Exosomal RNAs were extracted from 1 ml of urine using the exoRNeasy midi kit (QIAGEN, Hilden, Germany) through spin column-based extraction following the manufacturer's protocols. The extracted exosomal RNAs were stored at − 80 °C until use. The expression of miRNAs was measured using quantitative real-time reverse transcriptase PCR (qRT-PCR) with human TaqMan miRNA (Applied Biosystems, Foster City, CA). Complementary DNA (cDNA) was synthesized using the TaqMan miRNA Reverse Transcription Kit (Applied Biosystems, Foster City, CA), and qRT-PCR was performed using TaqMan miRNA-specific primers (assay IDs: hsa-miR-29a-3p:478587; hsa-miR-29c-3p:479229; hsa-miR-29b-3p:478369; hsa-miR-126-3p:477887; hsa-miR-26b-3p:483077; hsa-miR-124-3p:480901; hsa-miR-615-3p:478175; hsa-miR-199a-3p:477961; hsa-miR-199b-3p:478486; hsa-miR-335-3p:478033; hsa-miR-16-5p:477860, Applied Biosystems, Foster City, CA). The qRT-PCR reaction contained 1 μl of cDNA, 1 × TaqMan Universal PCR master mix, AmpErase Uracil N-Glycosylase, and 1 μl of primer mix (Applied Biosystems, Foster City, CA). Gene expression was measured using the ABI StepOnePlus real-time PCR system (Applied Biosystems). The reaction conditions were 10 min at 95 °C, followed by 40 cycles of 15 s at 95 °C and 60 s at 60 °C. The relative miRNA expression levels were calculated using the 2^−∆∆Ct^ method. The values were expressed relatively to urine creatinine concentration, and then were log transformed (log copies/mg Cr).

### Urinary miRNA targets in a view of bioinformatics

Urinary miRNA target genes and biological pathways of the dysregulated miRNAs were identified using bioinformatics tools such as MiRWalk (http://mirwalk.umm.uni-heidelberg.de/), and TargetScan (http://www.targetscan. org/vert_72/), and miRDB (http://www.mirdb.org/). Pathway speculation of the dysregulated miRNAs was performed using DAVID (https://david.ncifcrf.gov/). The Cytoscape (version 3.9.1) software (https://cytoscape.org/) and Metascape (https://metascape.org/gp) were used for further analysis^14^.

### Statistical analyses

Baseline characteristics and clinical parameters of the study population are expressed as the mean ± standard deviation or as the number of patients and percentage. We analyzed the levels of urinary miRNAs after log_10_ transformation, as these levels of miRNAs were not normally distributed. Continuous variables were compared using the Kruskal–Wallis test for overall disease subgroup comparisons and the Mann–Whitney test for comparisons between two groups, whereas categorical variables were compared using the Pearson χ^2^-test. Error bars represent mean ± standard error. Spearman’s correlation analyses were used to compare urinary exosomal miRNA levels with eGFR and uPCR. Additionally, we performed Spearman’s correlation analysis between selected 11 urinary exosomal miRNAs in IgAN patients. The Cox proportional hazards model was used in the univariate analyses to assess the probability of disease progression. Receiver operating characteristic (ROC) curves were generated, and areas under the curve (AUC) were calculated to evaluate the prognostic performance of miRNAs in patients with IgAN comparing to IIgANRPT. All statistical analyses were performed using SPSS for Windows (version 22.0; IBM, Armonk, NY, USA), and graphs were generated using GraphPad Prism 8.0 and R studio. Statistical significance was defined as a two-sided P < 0.05 for all analyses.

### Ethics approval and consent to participate

All the study procedures were conducted in compliance with the ethical guidelines of the Declaration of Helsinki and were approved by the Institutional Review Board. The approval number from Kyung Hee University Hospital was 2022-12-040, and written informed consent was obtained from all participants.

## Results

### Baseline clinical characteristics of the participants

The baseline characteristics and laboratory results of the participants are shown in Table 1, and they were classified into IgAN, disease control, and normal control groups. Comparing with the normal control group, the IgAN group was older (44.5 ± 16.4 vs. 34.3 ± 17.6 years old, P = 0.043), had a higher prevalence of hypertension (HTN) (46.2 vs. 5.3%, P = 0.004), lower eGFR (69.7 ± 37.3 vs. 114.1 ± 22.8 mL/min/1.73 m^2^, P < 0.0001), and higher uPCR (2.4 ± 2.7 vs. 0.1 ± 0.1 g/gCr, P < 0.0001).

**Table 1:** Baseline characteristics of the study population.

	Total (n = 123)	IgAN (n = 93)	MGN (n = 11)	Normal control (n = 19)	P
Age (years)	43.4 ± 17.1	44.5 ± 16.4^c^	49.9 ± 18.1^c^	34.3 ± 17.6^ab^	0.043
Male (n, %)	67(54.5)	47(50.5)	8(72.7)	12(63.2)	0.268
HTN (n, %)	48(39.0)	43(46.2)^c^	4(36.4)^c^	1(5.3)^ab^	0.004
DM (n, %)	5(4.1)	5(5.4)	0(0.0)	0(0.0)	0.431
BMI (kg/m^2^)	23.7 ± 2.9	24.0 ± 3.0^c^	23.3 ± 1.1	22.3 ± 2.5^a^	0.081
Albumin (g/dL)	3.8 ± 0.6	3.7 ± 0.6^c^	3.6 ± 0.8^c^	4.4 ± 0.3^ab^	< 0.0001
Creatinine (mg/dL)	1.4 ± 1.2	1.6 ± 1.3^bc^	0.7 ± 0.1^a^	0.8 ± 0.2^a^	< 0.0001
eGFR (ml/min/1.73 m^2^)	80.1 ± 38.5	69.7 ± 37.3^bc^	109.3 ± 15.7^a^	114.1 ± 22.8^a^	< 0.0001
C3 (mg/dL)	107.5 ± 19.9	108.3 ± 20.4	113.3 ± 26.3	100.4 ± 10.8	0.164
IgA (mg/dL)	304.4 ± 106.3	334.5 ± 99.2^bc^	213.7 ± 57.0^a^	206.2 ± 67.4^a^	< 0.0001
Urine PCR (g/gCr)	2.1 ± 2.7	2.4 ± 2.7^c^	2.9 ± 3.6^c^	0.1 ± 0.1^ab^	< 0.0001

IgAN, IgA nephropathy; MGN, membranous glomerulonephritis; BMI, body mass index; HTN, hypertension; DM, diabetes mellitus; eGFR, estimated glomerular filtration rate; PCR, protein-creatinine ratio; RBC, red blood cell; HPF, high-power field.

a: P < 0.05, vs. IgAN; b: P < 0.05, vs. MGN; c: P < 0.05, vs. normal control.

**Figure 2:**
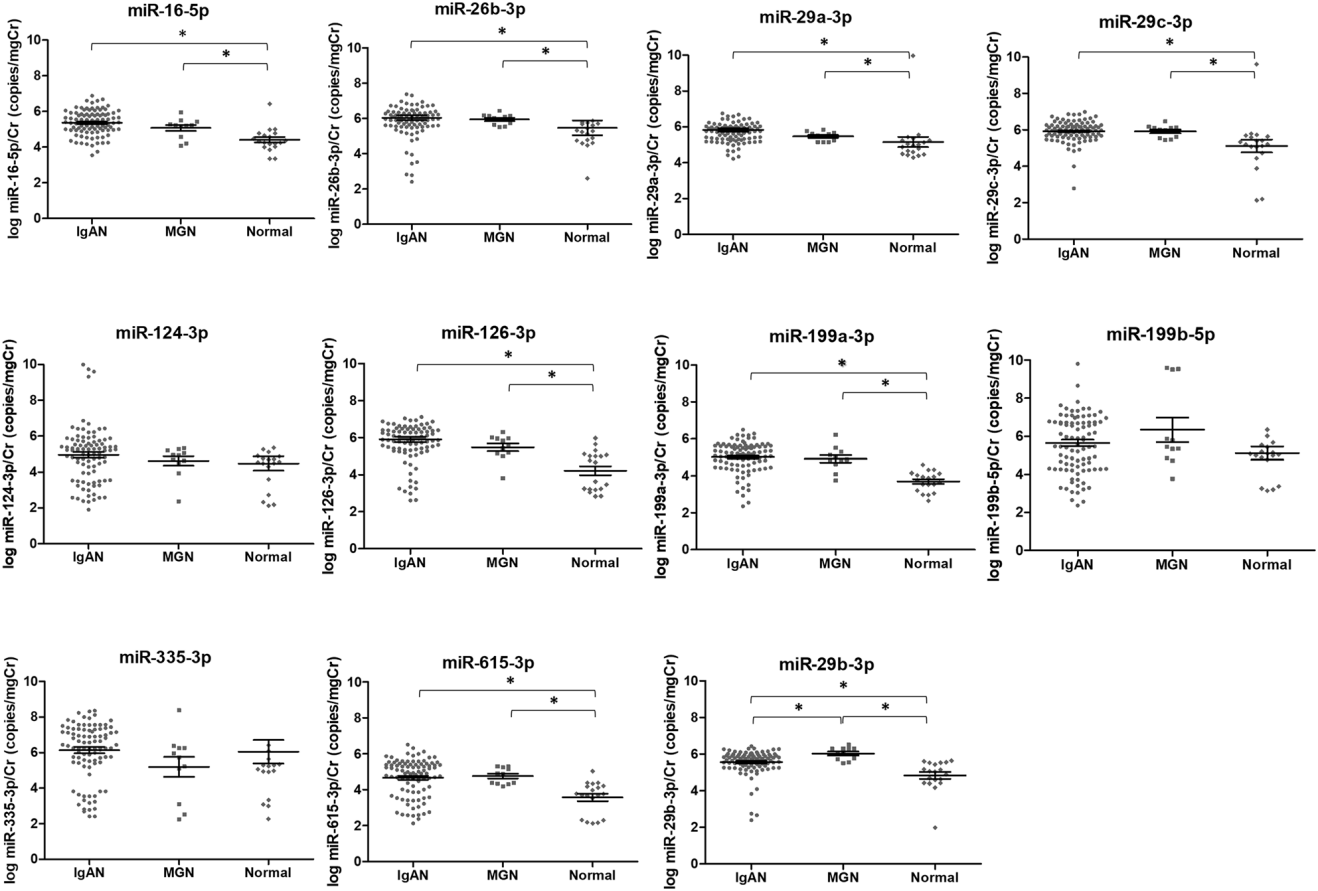
The levels of urinary exosomal miRNAs according to IgAN, MGN, and normal control group.

Urinary exosomal miR-16-5p, miR-26b-3p, miR-29a-3p, miR-29c-3p, miR-126-3p, miR-199a-3p, miR-615-3p, and miR-29b-3p levels were dysregulated in the IgAN group compared to those in the normal control group (Fig. 2). The expression level of miR-29b-3p in the IgAN group was significantly lower than that in the MGN group (Fig. 2).

### Correlations of urinary exosomal miRNAs with clinicopathological factors in IgAN patients

**Figure 3:**
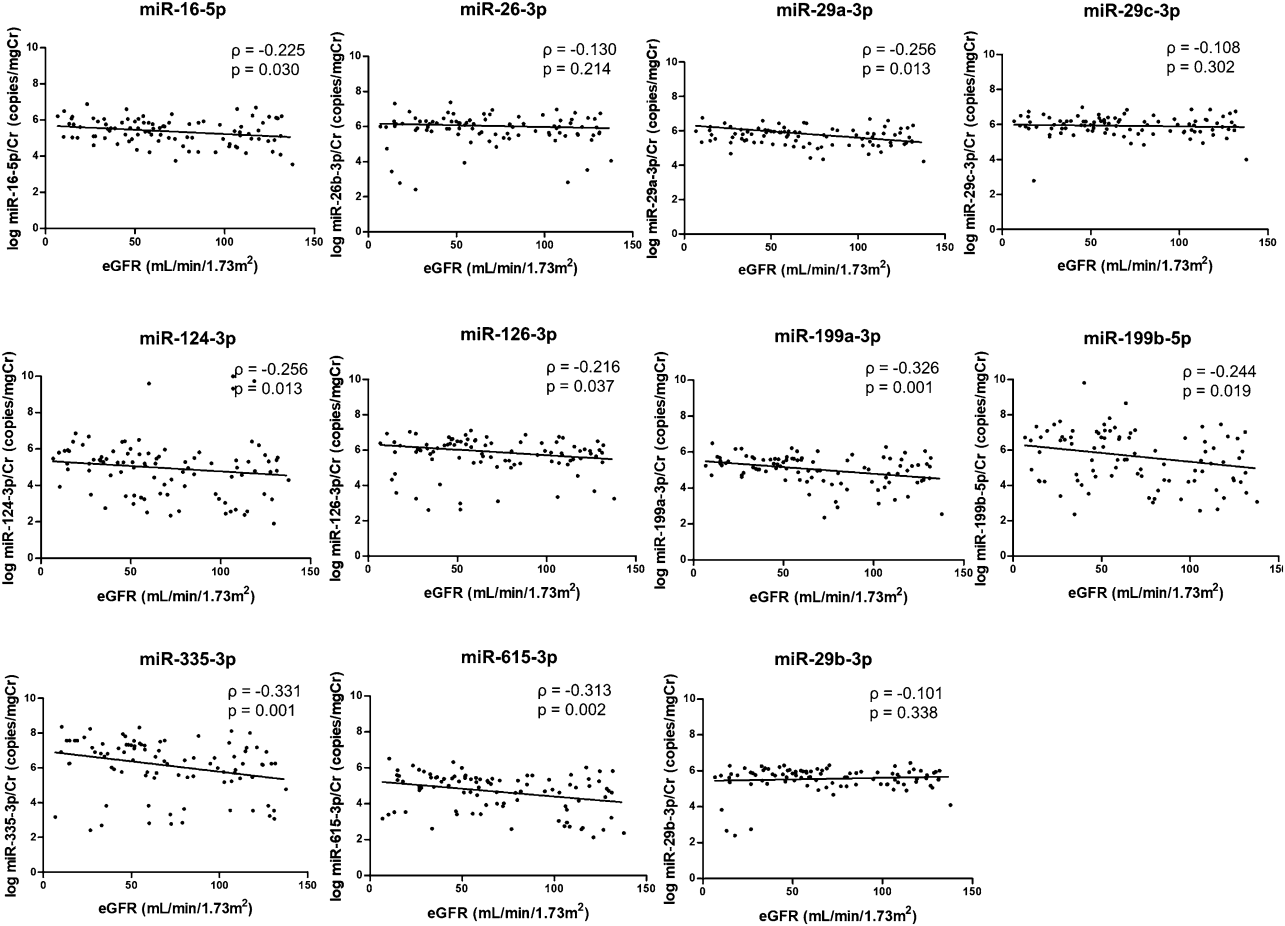
Correlation between the urinary exosomal miRNA level and eGFR in the patients with IgAN.

**Figure 4:**
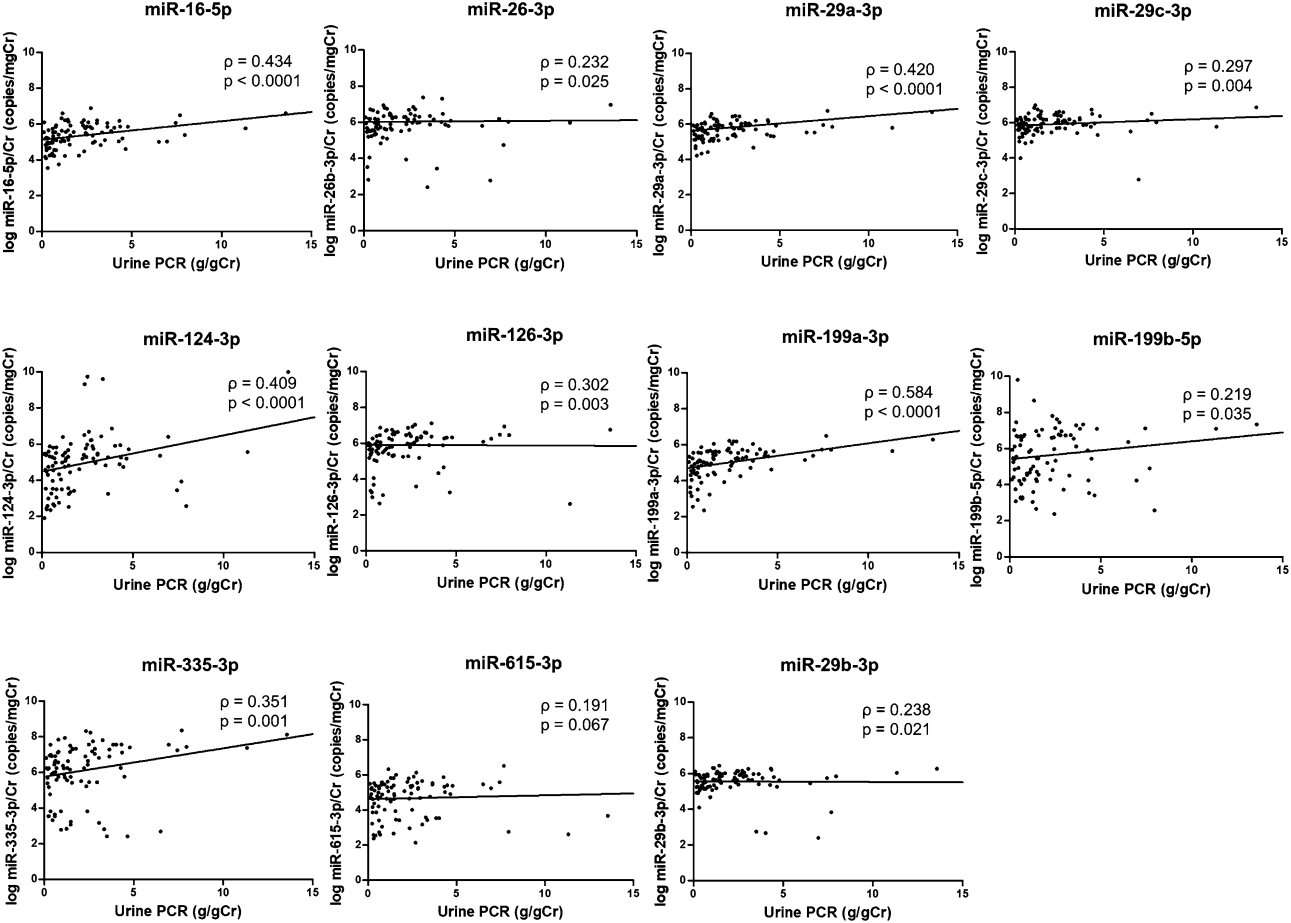
Correlation between the urinary exosomal miRNA level and urine protein-to-creatinine ratio in the patients with IgAN.

Figure 3 shows the negative correlation between the levels of most urinary exosomal miRNAs (miR-16-5p, miR-29a-3p, miR-124-3p, miR-126-3p, miR-199a-3p, miR-199b-5p, miR-335-3p, and miR-615-3p) and eGFR in IgAN patients. The levels of 10 selected urinary exosomal miRNA showed a positive correlation with uPCR, except for miR-615-3p (Fig. 4).

Urinary exosomal miR-26b-3p, miR-29a-3p, miR-29c-3p, miR-199a-3p, miR-199b-5p, miR-335-3p, and miR-29b-3p levels were significantly elevated in patients with IgAN who had mesangial hypercellularity (M0 vs. M1), according to the Oxford classification (Table 2). Among the 11 urinary exosomal miRNAs, an increase in miR-16-5p and miR-199a-3p levels was significant in patients with endocapillary hypercellularity (E0 vs. E1). Two miRNA levels, miR-26-3p and miR-29b-3p, were significantly elevated in patients with segmental glomerulosclerosis (S0 vs. S1). Finally, miR-126b-3p and miR-615-3p levels were significantly elevated in patients with crescents (C0 vs. C1-2) according to the Oxford classification.

**Table 2:** Urinary exosomal miRNA levels according to pathological findings based on the Oxford classification (MEST-C) in patients with IgAN.

Oxford classification	M			E			S			T			C		
score	0	1	P	0	1	P	0	1	P	0	1,2	P	0	1,2	P
n (%)	33 (35.5)	60 (64.5)		73 (78.5)	20 (21.5)		60 (64.5)	33 (35.5)		64 (68.8)	29 (31.2)		70 (75.3)	23 (24.7)	
miR-16-5p*	5.19 ± 0.76	5.47 ± 0.65	0.076	5.29 ± 0.72	5.66 ± 0.55	0.021	5.35 ± 0.74	5.42 ± 0.61	0.866	5.37 ± 0.65	5.37 ± 0.82	0.746	5.30 ± 0.71	5.60 ± 0.62	0.092
miR-26b-3p*	5.65 ± 0.97	6.25 ± 1.47	0.020	5.86 ± 1.15	6.69 ± 1.78	0.169	5.67 ± 0.98	6.71 ± 1.64	0.001	6.11 ± 1.48	5.88 ± 0.96	0.715	6.07 ± 1.46	5.95 ± 0.91	0.418
miR-29a-3p*	5.46 ± 0.57	6.03 ± 1.13	0.002	5.75 ± 0.94	6.11 ± 1.18	0.056	5.76 ± 1.06	5.94 ± 0.89	0.121	5.90 ± 1.12	5.66 ± 0.67	0.721	5.84 ± 1.12	5.79 ± 0.48	0.413
miR-29c-3p*	5.81 ± 0.60	5.98 ± 0.59	0.046	5.88 ± 0.63	6.07 ± 0.40	0.140	5.85 ± 0.67	6.05 ± 0.40	0.232	5.92 ± 0.59	5.92 ± 0.61	0.607	5.87 ± 0.65	6.06 ± 0.38	0.213
miR-124-3p*	4.84 ± 1.45	5.01 ± 1.63	0.465	4.95 ± 1.54	4.98 ± 1.66	0.874	4.83 ± 1.52	5.17 ± 1.62	0.238	4.98 ± 1.76	4.90 ± 1.02	0.901	5.02 ± 1.69	4.76 ± 1.07	0.551
miR-126-3p*	5.69 ± 1.30	6.02 ± 1.51	0.116	5.94 ± 1.46	5.75 ± 1.38	0.282	5.72 ± 1.24	6.24 ± 1.71	0.180	5.84 ± 1.48	6.04 ± 1.36	0.131	5.67 ± 1.37	6.62 ± 1.43	0.003
miR-199a-3p*	4.73 ± 0.90	5.18 ± 0.75	0.010	4.93 ± 0.84	5.37 ± 0.72	0.023	4.98 ± 0.89	5.10 ± 0.72	0.676	5.01 ± 0.71	5.04 ± 1.07	0.270	4.94 ± 0.85	5.26 ± 0.74	0.091
miR-199b-5p*	5.18 ± 1.30	5.90 ± 1.81	0.046	5.59 ± 1.66	5.84 ± 1.78	0.575	5.55 ± 1.68	5.82 ± 1.69	0.352	5.68 ± 1.77	5.58 ± 1.46	0.908	5.47 ± 1.68	6.20 ± 1.56	0.071
miR-335-3p*	5.39 ± 2.15	6.55 ± 1.28	0.0005	6.11 ± 1.70	6.25 ± 1.83	0.364	5.98 ± 1.91	6.43 ± 1.29	0.244	6.20 ± 1.66	6.01 ± 1.88	0.715	6.17 ± 1.68	6.04 ± 1.88	0.852
miR-615-3p*	4.68 ± 0.99	4.66 ± 1.09	0.773	4.61 ± 1.03	4.88 ± 1.14	0.172	4.70 ± 1.10	4.60 ± 0.98	0.413	4.68 ± 1.00	4.63 ± 1.18	0.881	4.54 ± 1.07	5.06 ± 0.92	0.029
miR-29b-3p*	5.44 ± 0.66	5.63 ± 0.72	0.022	5.52 ± 0.75	5.69 ± 0.50	0.274	5.44 ± 0.81	5.78 ± 0.37	0.020	5.61 ± 0.66	5.45 ± 0.79	0.460	5.57 ± 0.68	5.54 ± 0.77	0.936

M, mesangial hypercellularity; E, endocapillary hypercellularity; S, segmental glomerulosclerosis; T, interstitial fibrosis/tubular atrophy; C, crescents; miR, microRNA.

*Biomarker values are expressed relative to urine creatinine concentration and then log-transformed.

### Correlation between selected 11 urinary exosomal miRNAs in IgAN patients

The majority of miRNAs showed statistically significant positive Spearman correlations with each other (Supplementary Fig. 1). In the case of the Spearman correlation with miR-199b-5p, there were a few miRNAs that did not reach statistical significance. Urinary miR-199a-3p and miR-16-5p showed very strong Spearman correlation coefficient. Other very strong Spearman correlations were observed between miR-16-5p and miR-29c-3p as well as miR-29c-3p and miR-29a-3p.

### Urinary exosomal miRNAs for prediction of disease progression in patients with IgAN

Twenty-one patients in the IgAN group progressed to CKD, while 34 patients were classified as non-progressors based on the definition of disease progression in this study (Table 3). Progressors exhibited a higher prevalence of HTN (P = 0.002), lower eGFR (P < 0.0001), and higher uPCR (P < 0.0001). There was no significant difference between the two groups in the use of angiotensin II receptor blockers, angiotensin-converting enzyme inhibitors, or immunosuppressants. Mesangial (P = 0.028) and endocapillary (P = 0.047) hypercellularity were more frequently found in the disease progression group.

**Table 3:** Clinical characteristics of the patients with IgAN according to disease progression.

	Progression (n = 21)	Non-progression (n = 34)	P
Age (years)	51.6 ± 16.5	40.7 ± 15.4	0.020
Male (n, %)	14 (66.7)	13 (38.2)	0.041
HTN (n, %)	16 (76.2)	12 (35.3)	0.002
DM (n, %)	1 (4.8)	2 (5.9)	0.859
BMI (kg/m^2^)	25.2 ± 3.5	23.4 ± 2.9	0.052
Albumin (g/dL)	3.5 ± 0.5	3.8 ± 0.6	0.156
IgA (mg/dL)	309.4 ± 88.8	334.3 ± 109.5	0.905
eGFR (ml/min/1.73 m^2^)	33.1 ± 26.0	79.1 ± 34.6	< 0.0001
Urine PCR (g/gCr)	3.8 ± 2.4	1.9 ± 2.6	< 0.0001
Use of ARB or ACEi (n, %)	18 (85.7)	26 (76.5)	0.394
Use of immunosuppressant (n, %)	19 (90.5)	24 (70.6)	0.059
Oxford classification M (n, %)			0.028
0	4 (19.0)	16 (47.1)	
1	17 (81.0)	18 (52.9)	
Oxford classification E (n, %)			0.047
0	15 (71.4)	32 (94.1)	
1	6 (28.6)	2 (5.9)	
Oxford classification S (n, %)			0.742
0	12 (57.1)	21 (61.8)	
1	9 (42.9)	13 (38.2)	
Oxford classification T (n, %)			0.155
0	12 (57.1)	26 (76.5)	
1, 2	9 (42.9)	8 (23.5)	
Oxford classification C (n, %)			0.070
0	13 (61.9)	29 (85.3)	
1, 2	8 (38.1)	5 (14.7)	

BMI, body mass index; HTN, hypertension; DM, diabetes mellitus; eGFR, estimated glomerular filtration rate; PCR, protein creatinine ratio; ARB, angiotensin II receptor blockers; ACEi, angiotensin II converting enzyme inhibitors; M, mesangial hypercellularity; E, endocapillary hypercellularity; S, segmental glomerulosclerosis; T, interstitial fibrosis/tubular atrophy; C, crescents.

**Figure 5:**
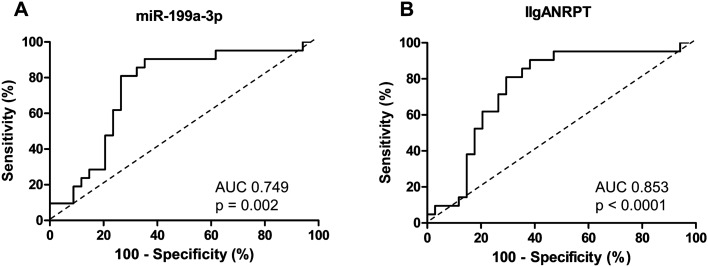
ROC analysis of urinary miRNA expression in distinguishing “progressors” among patients with IgAN. Each panel represents ROC analysis: (**A**) miR-199a-3p, and (**B**) IIgANRPT.

In the univariate analysis, age, BMI, HTN, eGFR, uPCR, Oxford classification E, and three miRNAs (miR-16-5p, miR-199a-3p, and miR-335-3p) were statistically significant in disease progression of IgAN (Table 4). Among the selected miRNAs in this study, miR-199a-3p showed the lowest p-value (P = 0.009). To assess the predictive value of miRNAs on disease progression, ROC curves were generated for miR-199a-3p, and IIgANRPT in patients with IgAN. The AUC results of miR-199a-3p were 0.749 (95% CI 0.614–0.884, P < 0.0001) in Fig. 5. The AUC of IIgANRPT per se was 0.853 (95% CI 0.749–0.957, P < 0.0001). ROC curves combining miR-199a-3p with the other two miRNAs that exhibited statistical significance are presented in Supplementary Fig. 2.

**Table 4:** Predictors of disease progression in univariate Cox regression analysis in patients with IgAN.

Variables	Univariate analysis	
	HR (95% CI)	P
Age	1.03 (1.01–1.06)	0.012
Male	2.26 (0.91–5.60)	0.078
HTN	3.87 (1.42–10.57)	0.008
DM	1.42 (0.19–10.63)	0.734
BMI	1.13 (1.00–1.27)	0.048
IgA	1.00 (0.99–1.00)	0.433
eGFR	0.96 (0.95–0.98)	< 0.0001
Urine PCR	1.13 (1.02–1.24)	0.015
Use of ARB or ACEi	1.14 (0.34–3.88)	0.831
Use of immunosuppressant	2.46 (0.57–10.55)	0.226
Oxford classification M		
0	1	
1	2.70 (0.91–8.02)	0.074
Oxford classification E		
0	1	
1	4.16 (1.59–10.88)	0.004
Oxford classification S		
0	1	
1	1.23 (0.52–2.93)	0.634
Oxford classification T		
0	1	
1, 2	1.76 (0.74–4.18)	0.199
Oxford classification C		
0	1	
1, 2	2.26 (0.94–5.45)	0.070
miR-16-5p*	2.13 (1.14–3.98)	0.018
miR-26b-3p*	1.09 (0.76–1.55)	0.645
miR-29a-3p*	1.27 (0.99–1.64)	0.065
miR-29c-3p*	1.36 (0.52–3.52)	0.531
miR-124-3p*	1.23 (0.97–1.56)	0.082
miR-126-3p*	1.13 (0.82–1.54)	0.452
miR-199a-3p*	2.76 (1.29–5.89)	0.009
miR-199b-5p*	1.03 (0.78–1.37)	0.839
miR-335-3p*	1.31 (1.02–1.68)	0.035
miR-615-3p*	1.05 (0.69–1.61)	0.819
miR-29b-3p*	0.97 (0.56–1.67)	0.906

HR, hazard ratio; CI, confidence interval; BMI, body mass index; HTN, hypertension; DM, diabetes mellitus; eGFR, estimated glomerular filtration rate; PCR, protein creatinine ratio; ARB, angiotensin II receptor blocker; ACEi, angiotensin II converting enzyme inhibitor; M, mesangial hypercellularity; E, endocapillary hypercellularity; S, segmental glomerulosclerosis; T, interstitial fibrosis/tubular atrophy; C, crescents; miR, microRNA.

*Biomarker values are expressed relative to urine creatinine concentration and then log-transformed.

### Target gene prediction and enrichment pathway analysis of dysregulated urinary exosomal miRNAs

Further studies to determine the pathogenesis of IgAN were performed using bioinformatics through the regulation of target genes and pathways. We predicted target genes for each of the three miRNAs (miR-16-5p, miR-199a-3p, and miR-335-3p) based on the intersection of the miRWalk, TargetScan, and miRDB databases, respectively (Supplementary Fig. 3). A total of 123 miR-16-5p and 37 miR-199a-3p target genes shared four target genes. Seven target genes were common between 123 miR-16-5p and 139 miR-335-3p target genes. One target gene was found between 37 miR-199a-3p and 139 miR-199a-3p.

Since miR-199a-3p showed an overwhelming number of genes and had one of the most powerful p-values, further analysis was conducted on miR-199a-3p. MiR-199a-3p related enrichment clusters included the cell–cell adhesion, regulation of angiogenesis, protein dephosphorylation or phosphorylation, and so on (Supplementary Fig. 4). Considerable bioinformatic connections between various enrichment pathways and miRNAs are being examined.

## Discussion

In this study, we explored the expression levels and roles of 11 urinary exosomal miRNAs in the early diagnosis of progressive IgAN. The expression levels of five miRNAs (miR-16-5p, miR-199a-3p, and miR-335-3p) were significantly associated with disease progression in the IgAN group and were closely correlated with eGFR and uPCR. The expression levels of urinary exosomal miRNAs have been examined as surrogates of clinical prognosis in IgAN patients^12,15^. This study aimed to differentiate IgAN-specific urinary miRNAs relavent to disease progression with favorable discrimination results.

The significance of the five miRNAs that demonstrated statistically significant results in the univariate analysis, as well as their predicted target genes, has been reported in multiple previous studies. In this study, miR-199a-3p was the leading urinary exosomal miRNA in terms of its prognostic value for clinical outcomes. MiR-199a-3p is involved in cell–cell adhesion, angiogenesis, and proliferation in various diseases^16,17^. MiR-16-5p plays essential roles in bicarbonate transporters, the response to hypoxia, and autophagy according to enrichment analysis. Furthermore, a recent article suggested urinary miR-16-5p as a promising biomarker of endocapillary hypercellularity for IgAN patients^18^. Lastly, upregulated miR-335-3p is known to prevent aging related to cell cycle arrest, immune inflammation, and antioxidative enzymes, specifically in kidney mesangial cells, and the result is consistent with the correlation found in M-lesions of the Oxford Classification^19,20^. Through IgAN-targeted further research, miRNAs could be applied to predict disease prognosis and could even be a target for disease treatment.

This study is valuable as we conducted RNA sequencing in a meta-analysis and predicted miRNA candidates using RNAs. To be specific, significant miRNAs could be identified since IgAN-specific miRNAs were predicated after selecting IgAN-specific RNAs in human-based database through meta-analysis^21,22^. Then, these 11 miRNAs were validated in urinary exosomes as being evaluated for their clinical predictive values in patients with IgAN. Second, we attempted to elucidate the correlation between the expression levels of miRNAs and clinical and pathological parameters, which are already recognized in clinical practice as disease progression-related features. Additionally, miRNA candidates were powerful in that they showed an AUC of 0.786 without invasive renal biopsy-related information. Throughout the result of this study, we could compare each selected urinary biomarker and clinicopathological characteristic of patients with IgAN on predictive function of clinical outcomes.

Despite these strengths, this study had several limitations. First, we included a relatively small number of participants. Two control groups were relatively much smaller than the IgAN group. The limited sample size was attributed to the collection of urine samples from patients with biopsy-proven IgAN, MGN, and normal controls. Despite the limitation in the number of participants, this enabled clear differentiation of each patient's phenotype and pathology, which is an advantage of our study. Consequently, validating these results in large-scale cohorts is necessary. Second, some recent GSE datasets reflecting the progression over time were not incorporated into our study. The 10 GSE datasets that formed the basis of our research were constructed up until the early months of 2020, excluding any datasets registered after the second half of 2020. We investigated the relevance of findings from some GSE datasets, including GSE141295 and GSE141344, which were relatively recently released, to our own research results^23,24^. Subsequently, we conducted additional analysis using the GSE141295 dataset to compare significant miRNAs with our findings. Out of the 11 selected miRNAs, six were found to be statistically significant (Supplementary Table 4). From the GSE141344 dataset, none of the 11 miRNAs selected through our prediction were found to be statistically significant. While our analysis primarily focused on comparing IgA patients with normal controls in miRNA prediction, the absence of a normal control group in this dataset may be one of the reasons for not finding significant results in the comparison. Continuous updates and follow-up will be necessary in future studies to address this limitation. Third, the levels of urinary miRNAs were measured only once, at the time of the renal biopsy, which may result in an incorrect correlation of parameters. Urinary miRNAs may have under or overestimated kidney function because of a one-time laboratory checkup. Fourth, the expression levels of miRNAs were relatively analyzed to urine creatinine concentration (log copies/mg Cr), however, differences in exosome quantity in the urine may contribute to the urinary expression levels of miRNA. Fifth, disease progression, defined as a 50% decline in eGFR or condition in need of renal replacement therapy within five years, may lead to under- or overestimation of worsening clinical outcomes. To mitigate differences in clinical progression due to longer follow-up periods, we adopted the similar definition on progression proposed in previous studies^18,24–26^. However, this study did not have a significantly longer follow-up period compared to other studies; thus, subsequent research is necessary to fully explore this aspect. Sixth, different therapeutic strategies could decline the kidney function in patients with IgAN, who were prescribed RAS blockers or immunosuppressive drugs such as corticosteroids when they met the criteria for treatment options. Accordingly, therapeutic strategies were adjusted to analyze clinical outcomes. Lastly, although the premise of identified miRNAs in patients with IgAN is valid, it can be argued that these identified miRNAs could be found in patients with CKD or other glomerulonephritis diseases. While it could be challenging to distinguish IgAN from other kidney diseases, the most clinically significant point is predicting disease progression in IgAN. Through clinical approach individual or combined miRNA candidates along with the existing IIgANRPT, it would help to more aggressively select patients with IgAN for immunosuppressive therapy and other treatments.

## Conclusion

This study reported high levels of miRNA in urinary exosomes of patients with IgAN. In addition, it found that urinary exosome levels of miR-16-5p, miR-199a-3p, and miR-335-3p were significantly associated with disease progression of IgAN. Furthermore, urinary exosomal miRNAs like miR-199a-3p may serve as non-invasive biomarkers for the early detection of disease progression. As early diagnostic biomarkers play a key role in predicting renal outcomes, ongoing studies are warranted to clarify urinary exosomal miRNAs as indicators of renal damage in patients with IgAN.

### Supplementary Information

Supplementary Information.

Supplementary Table 2.

Supplementary Table 3.

Supplementary Table 4.

## Data Availability

The data underlying this article will be shared by the corresponding author upon reasonable request.
