# Supplementary material for: Clinical significance of urinary exosomal microRNAs in patients with IgA nephropathy
Source: Sci Rep. 2023 Oct 11;13:17201. doi: 10.1038/s41598-023-44460-5 (PMC10567798; doi:10.1038/s41598-023-44460-5)

Clinical Significance of Urinary Exosomal microRNAs in patients with IgA nephropathy

Soo-Young Yoon[1], Jin Sug Kim[1], Su Woong Jung[2], Yang Gyun Kim[2], Hyeon Seok Hwang[1], Ju Young Moon[2], Sang-Ho Lee[2], Jung-Woo Seo[3], Junhee Seok[4], Donghyun Tae[4], Kyunghwan Jeong[1*]

Supplementary table 1. Information on 10 IgAN GSE datasets (consisting of five datasets for the glomerulus and five for the tubulointerstitium). DN, diabetic nephropathy; FSGS, focal segmental glomerulosclerosis; IgAN, IgA nephropathy; MGN, membranous glomerulonephritis; MCD, minimal change disease; RPGN, rapidly progressive glomerulonephritis; SLE, systemic lupus erythematosus; TMD, thin basement membrane disease.

Supplementary table 2. Significant gene lists from the glomerulus datasets obtained through microarray analysis. When the FDR value is displayed as 0, it corresponds to values less than 0.0000161.

abs, absolute; FDR, false discovery rate; FC, fold change.

Supplementary table 3. Significant gene lists from the tubulointerstitium datasets obtained through microarray analysis. When the FDR value is displayed as 0, it corresponds to values less than 0.00000104.

abs, absolute; FDR, false discovery rate; FC, fold change.

Supplementary table 4. Significant miRNAs among the 11 urinay miRNAs using the GSE141295 dataset.

Supplementary figure 1. Spearman correlation analysis of the 11 urinary miRNAs. Significant correlations ($p < 0.05$) are represented by the Spearman correlation coefficient between two miRNAs, while non-significant correlations are indicated with "x". The legend shows the color range of different Spearman correlation coefficient values.

Supplementary figure 2. ROC analysis of urinary miRNA expression in distinguishing "progressors" among patients with IgAN. Each panel represents ROC analysis: **A** miR-16-5p*miR-199a-3p, and **B** miR-199a-3p*miR-355-3p.

Supplementary figure 3. Interaction sizes of target genes among three urinary miRNAs (miR-16-5p, miR-199a-3p, and miR-355-3p).

Supplementary figure 4. Network of enrichment analysis of miR-199a-3p.

Supplementary table 1. Information on 10 IgAN GSE datasets (consisting of five datasets for the glomerulus and five for the tubulointerstitium).

GSE no.	Title	sample type (Human kidney tissue)	Journal
**Glomerulus**			
GSE104948	Glomerular Transcriptome from European Renal cDNA Bank subjects and living donors	Glom DN, MCD, IgAN, MGN, SLE, FSGS, MCD, RPGN	Ann Rheum Dis. 2018 Aug;77(8):1226-1233
GSE93798	Transcriptomic and proteomic profiling reveal insights of mesangial cell function in patients with IgA Nephropathy	Glomeruar compart from control human kidney vs IgAN	J Am Soc Nephrol 2017 Oct;28(10):2961-2972
GSE99339	Transcriptome-based network analysis reveals renal cell type-specific dysregulation of hypoxia-associated transcripts	Glom DN, MCD, IgAN, MGN, SLE, FSGS, MCD, RPGN	Sci Rep 2017 Aug 17;7(1):8576
GSE50469	The molecular phenotype of endocapillary proliferation in IgA nephropathy and potential modulation by bioactive small molecules	glomerular transcriptome of microdissected kidney biopsies from 22 patients with IgAN	PLoS One 2014;9(8):e103413
GSE37460	Cross-species transcriptional network analysis defines shared inflammatory responses in murine and human lupus nephritis	healthy living donor , IgAN glom	J Immunol 2012 Jul 15;189(2):988-1001
**Tubulointerstitium**			
GSE104954	Tubulointerstitial transcriptome from ERCB subjects with chronic kidney disease and living donor biopsies.	Tub DN, MCD, TMD, IgAN, SLE, FSGS, MGN, LD, RPGN	Ann Rheum Dis 2018 Aug;77(8):1226-1233
GSE99340	Transcriptome-based network analysis reveals renal cell type-specific dysregulation of hypoxia-associated transcripts	Tub DN, MCD, TMD, IgAN, FSGS, MGN, SLE, RPGN	Sci Rep 2017 Aug 17;7(1):8576
GSE99325	Transcriptome-based network analysis reveals renal cell type-specific dysregulation of hypoxia-associated transcripts [Tub-FE]	Tub DN, MCD, TMD, IgAN, FSGS, MGN, SLE, RPGN	Sci Rep 2017 Aug 17;7(1):8576
GSE35488	Expression data from human with IgA nephropathy (IgAN) [HG-U133A_ENTREZG_10]	Tubulointerstitium IgAN, control living donor	PLoS One 2010 Oct 18;5(10):e13451
GSE35487	Expression data from human with IgA nephropathy (IgAN) [HG-U133A]	Tubulointerstitium IgAN, control living donor	PLoS One 2010 Oct 18;5(10):e13451

Supplementary figure 1. Spearman correlation analysis of 11 urinary miRNAs.

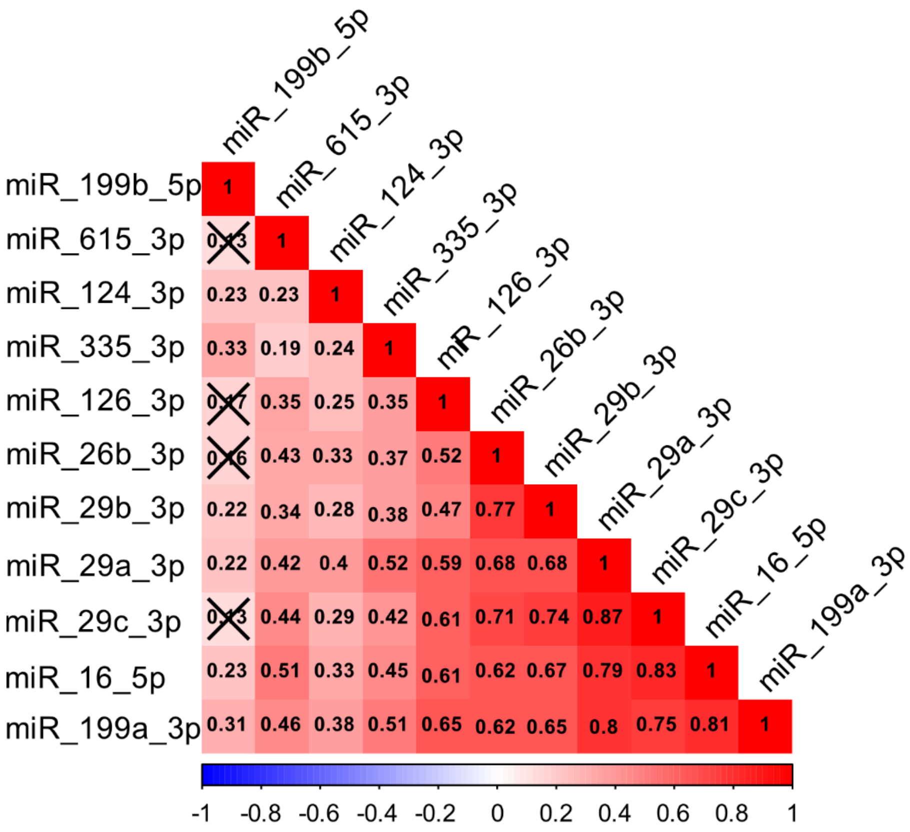

Supplementary figure 2. ROC analysis of urinary miRNA expression in distinguishing “progressors” among patients with IgAN.

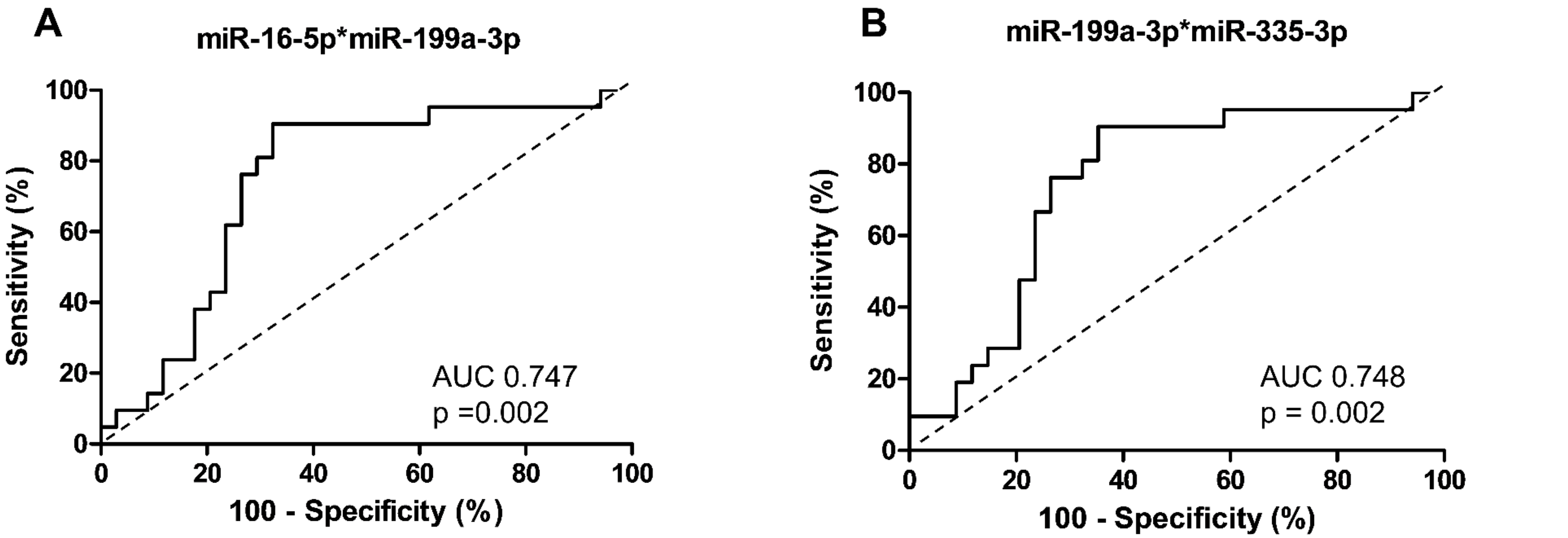

Supplementary figure 3. Interaction sizes of target genes among three urinary miRNAs (miR-16-5p, miR-199a-3p, and miR-355-3p).

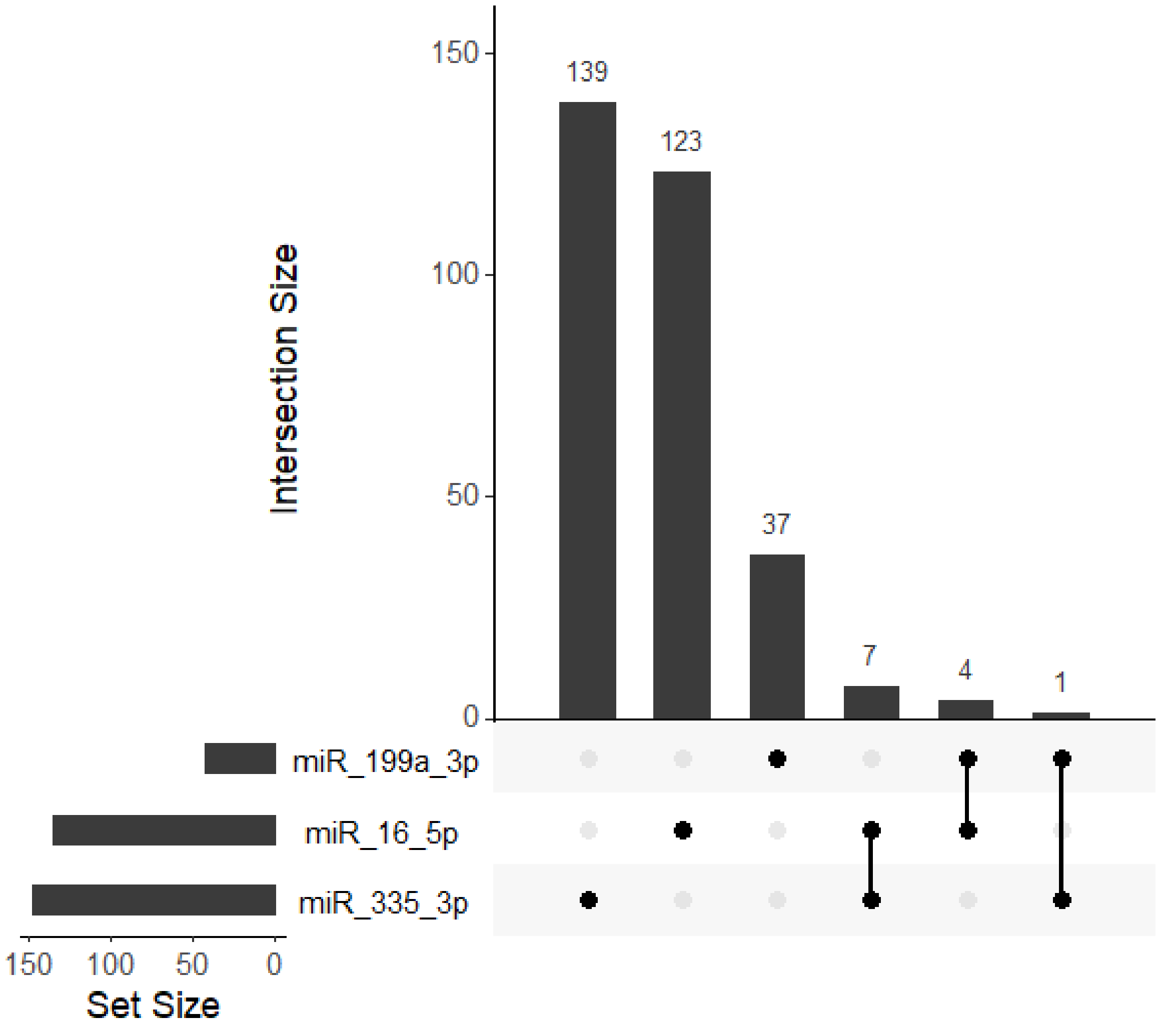

Supplementary figure 4. Network of enrichment analysis of miR-199a-3p.

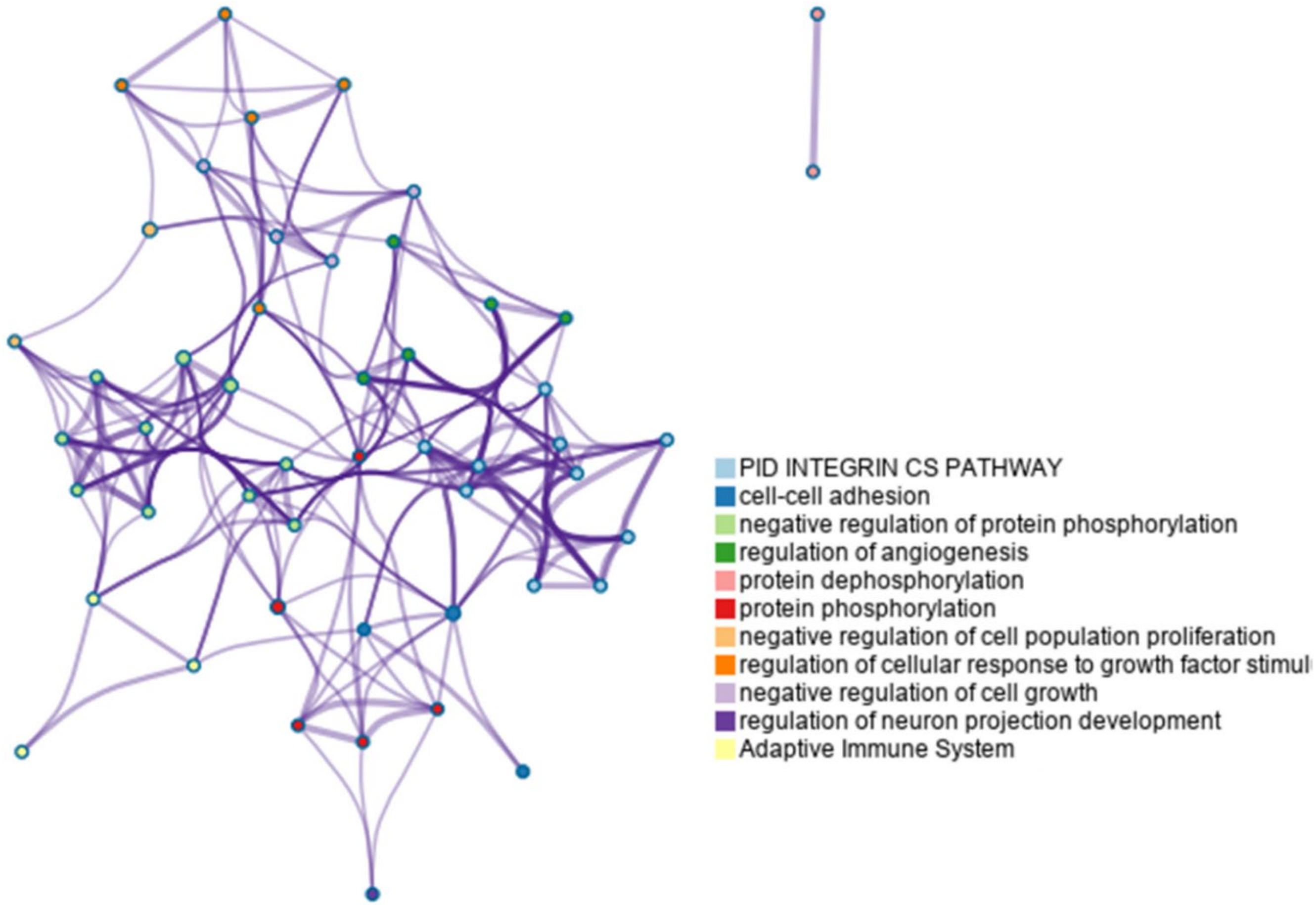

Supplement: Supplementary file 1 — Supplementary Information. [file 41598_2023_44460_MOESM1_ESM.pdf]
